# Isolation and no-entry marine reserves mitigate anthropogenic impacts on grey reef shark behavior

**DOI:** 10.1038/s41598-018-37145-x

**Published:** 2019-02-27

**Authors:** Jean-Baptiste Juhel, Laurent Vigliola, Laurent Wantiez, Tom B. Letessier, Jessica J. Meeuwig, David Mouillot

**Affiliations:** 1 Université de la Nouvelle-Calédonie, ISEA EA1314, BPR4, 98851 Noumea, New Caledonia; 2 Institut de recherche pour le développement (IRD), UMR ENTROPIE, Laboratoire Excellence LABEX Corail, Noumea, New Caledonia; 3 Université de Montpellier, CNRS, IRD, Ifremer, UMR MARBEC, 34095 Montpellier Cedex 5, France; 4 Institute of Zoology, Zoological Society of London, Regent’s Park, London, NW1 4RY UK; 5 Centre for Marine Futures, Oceans Institute and School of Animal Biology, The University of Western Australia, (M470), 35 Stirling Highway, Crawley, WA 6009 Australia; 6 Australian Research Council Centre of Excellence for Coral Reef Studies, James Cook University, Townsville, QLD 4811 Australia

## Abstract

Reef sharks are vulnerable predators experiencing severe population declines mainly due to overexploitation. However, beyond direct exploitation, human activities can produce indirect or sub-lethal effects such as behavioral alterations. Such alterations are well known for terrestrial fauna but poorly documented for marine species. Using an extensive sampling of 367 stereo baited underwater videos systems, we show modifications in grey reef shark (*Carcharhinus amblyrhynchos*) occurrence and feeding behavior along a marked gradient of isolation from humans across the New Caledonian archipelago (South-Western Pacific). The probability of occurrence decreased by 68.9% between wilderness areas (more than 25 hours travel time from the capital city) and impacted areas while the few individuals occurring in impacted areas exhibited cautious behavior. We also show that only large no-entry reserves (above 150 km²) can protect the behavior of grey reef sharks found in the wilderness. Influencing the fitness, human linked behavioral alterations should be taken into account for management strategies to ensure the persistence of populations.

## Introduction

Beyond overexploitation and the global defaunation of all ecosystems on Earth^1^, humans can induce behavioral alterations on animals with potential consequences on their fitness^2,3^. For instance, terrestrial wildlife like mammals^4^ and birds^5^ are more vigilant near humans, resulting in decreased food intake and reduced reproductive success. Similarly, marine mammals show behavioral alterations (*i.e*. decreased food intake and reduced reproductive success) due to nautical activities, resulting in additional energetic costs to avoid interactions or nuisance^6,7^. Sharks exhibit high learning abilities similar to most terrestrial vertebrates^8^. Behavior experiments demonstrate that sharks can learn discriminative tasks and retain information for long time periods^9,10^. These abilities make sharks also likely to demonstrate long-lasting adaptive responses to external stimuli including interactions with human activities^11^. For instance, the catchability of blacktip reef sharks (*Carcharhinus melanopterus*) decreases after several catches and releases^12^ while bull sharks (*Carcharhinus leucas*) show a modified behavior related to food provisioning^13^. Although human impacts on shark behavior have been documented, we still lack empirical evidence of behavioral alterations along a wide gradient of human activities from densely populated areas to the last wilderness areas.

Reef sharks are emblematic species providing socioeconomic benefits through tourism while playing multiple ecological roles in coastal ecosystems^14,15^. Their conservative life history traits such as slow growth rate, late sexual maturity and low fecundity, coupled with overfishing, induce severe population depletion worldwide and high extinction risk^16,17^. Consequently, reef sharks are of high conservation priority^18^. Marine reserves, a restrictive subset of marine protected areas^19^, are recognized as management tools to counteract human pervasive impacts by prohibiting extracting activities (no-take) or even human presence (no-entry)^20^. By lessening human pressure including extraction of their prey, marine reserves may attract sharks and restore their behavior under quasi-wilderness or undisturbed conditions^21^. However, only a very small portion of marine reserves are no-entry (around 6% worldwide)^19^ while the others tend to permit human activities even if non-destructive^22,23^. How marine reserves (no-take or no-entry) can be efficient management tools to preserve an unaltered reef shark behavior using wilderness areas as a benchmark is thus a critical but still unresolved question.

The grey reef shark (*Carcharhinus amblyrhynchos*) is a good model to evaluate the influence of human activities (including management) on shark occurrence and behavior since this species (*i*) is common and widely distributed across the entire Pacific region, (*ii*) is vulnerable to overfishing and prone to local extinction^18^, (*iii*) exhibits a highly inquisitive behaviour to novelty and disturbances as well as agonistic displays (*i.e*. intimidation behavior)^24^. The New Caledonian archipelago offers a unique opportunity to explore these relationships since (*i*) human population density is very heterogeneous along a marked gradient from the vicinity of the capital (179,509 inhabitants in Noumea from a total of 268,767 inhabitants in 2014) towards wilderness^25^, (*ii*) a wide range of restrictions have been set up from small (less than 5 km²) no-take reserves to long established (up to 44 years) and large (up to 172 km²) no-entry reserves, (*iii*) some areas are highly isolated from humans (more than 25 hours travel time away from Noumea) providing a fair benchmark to assess reserve effect on shark occurrence and behavior. Here we propose to test whether the occurrence and the behavior of grey reef sharks are altered along a gradient of isolation from humans. Moreover, we assess which type of marine reserves can prevent these alterations if any, using wilderness areas as reference conditions^26,27^.

## Material and Methods

### Study area

New Caledonia is an archipelago (21°30′S, 165°30′E) composed of a 400 km long main island (“Grande Terre”) longitudinally crossed by a mountainous chain that separates the east and west coasts, and a series of smaller islands. Historically, fisheries in New Caledonia have not targeted sharks, as these animals are totem for local populations. In addition, reported shark bycatch is negligible (27 kg/year on average from 2000 to 2013; Source: Government of New Caledonia) and a formal shark-fishing ban has been set in the Exclusive Economic Zone (EEZ) since 2013. Human population density is spatially distributed along a marked gradient from Noumea in the South-West, which hosts 67% of New Caledonia’s 268,767 inhabitants (2014), towards sparsely populated Northern parts of the main island and isolated wilderness coral reefs. New Caledonia also hosts diverse reserves including a series of small no-take reserves distributed along the isolation gradient from the capital city, an old large (150 km²) no-take close to the capital city (Aboré reef reserve) and one of the oldest (>40 years) and largest (172 km²) coral reef no-entry reserve (Merlet)^26^ (Table 1).

**Table 1:** Summary of marine reserves features sampled with stereo Baited Remote Underwater Video Stations (stereo-BRUVS) across the New Caledonian archipelago.

Reserve	Location	Status	Surface (km²)	Travel time range (h)	Creation year	N	% occurrence
Ténia islet	La Foa	No-take	10	1.32–1.77	1998	9	0.11
Ouano	La Foa	No-take	29.8	1.87–1.91	2004	5	0
Aboré reef	Nouméa	No-take	150	0.66–1.80	1981–96	21	38.1
Larégnère islet	Nouméa	No-take	6.7	0.41–0.47	1989	6	0
Hyabé-Le Jao	Pouébo	No-take	13.3	5.43–5.63	2010	19	0.05
Prony (2 sites)	Prony Bay	No-take	1.5	1.26–1.34	1993	12	0
(Yves) Merlet	Yaté	No-entry	172	1.61–3.26	1970	26	69.23
Beautemps –Beaupré	near Ouvéa	No-entry	125	16.68–18.74	Unknown	10	70

N represents the number of stereo-BRUVS deployments in the reserve.

### Video survey

**Figure 1:**
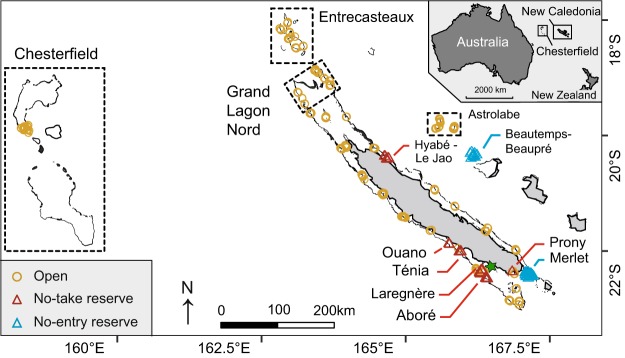
Map of the New Caledonian archipelago showing the deployment of stereo Baited Remote Underwater Video Stations (stereo-BRUVS) in marine reserves (triangles) and outside reserves (circles) to assess the occurrence and behavior of grey reef sharks. Wilderness areas are surrounded with dash lines and the capital city, Noumea, is symbolized by a green star. See Table 1 for the full list of reserves and their characteristics.

A total of 367 stereo-BRUVS were deployed on or near coral reefs of New Caledonia from September 2012 to October 2014 (Fig. 1): 108 in reserves (Table 1) and 259 in areas open to fishing including 114 in wilderness areas (above 25 hours of travel time from the capital city). Underwater video devices consisted of two HDR CX-7 or HDR CX-12 camcorders placed in sealed chambers and oriented horizontally towards the bait (1 kg of crushed pilchards, *Sardinops sagax*) with an inward convergent angle of 8°. The stereo Baited Remote Underwater Video Systems (stereo-BRUVS) were deployed from the boat at 15–20 m depth (mean ± SD: 15.9 ± 9.7 m) for one-hour recording. For each site, all the stereo-BRUVS were set at least 400 meters apart to consider each deployment as an independent replicate survey^28^. The probability of counting the same shark more than once was reduced because of the simultaneous deployment of adjacent stereo-BRUVS and because sharks remained a long time around the same device. Individuals already recorded and identified through morphometric and markings were removed from the dataset. Shark total length and distance from camera were measured using the EventMeasure software package (www.seagis.com.au).

### Behavioral responses to bait

Two traits of shark feeding behavior were recorded from each video. The first trait was the bite on the bait. It was coded 0 in the absence of bite and 1 when a grey reef shark bites the bait during video recording. The second trait was the approach towards the bait. It was measured from the entrance of the first individual in the field of view until it gets closer than 1 m from the bait. Approach type was coded from 0 to 2 according to the motivation for food intake and the wariness towards the Stereo-BRUVS. An approach coded 0 represented a repulsive or a neutral reaction with individuals swimming into the field of view of the cameras, but not towards the bait. An approach coded 1 represented a twisted approach with turns towards the bait. Individuals performing such approaches often turned around the device with many direction changes. A straight-line approach towards the bait was coded 2 and represented the highest level of motivation for food intake with no apparent wariness. Approach types were not measured at three stations due to difficulties in identifying and following the first individual. Stereo-BRUVS deployments were not repeated in time to avoid a putative adapting behavioral response. Individuals had never encountered Stereo-BRUVS devices before as no deployments were previously performed in New Caledonia.

### Explanatory variables

Five environmental variables (depth, time of the day, sea surface temperature (SST), coral reef habitat and coral reef area) and three human related variables (human population density, travel time from the capital city and the management of the sampled area), all known to be the primary predictors of change in coral reef ecosystems^16,29–31^ were considered to model grey reef shark occurrence and behavior. We built (i) Generalized Linear Models (GLM) using binomial distributions to explain both shark occurrence and bite, and (ii) Multinomial log-linear models via neural networks to explain the approach type which was coded as a multinomial variable. Each model considered all the eight explanatory variables detailed below.

Depth and time of the day were measured for each stereo-BRUVS deployment. Mean SST over the last 10 years was collected (www.podaac.jpl.nasa.gov). Reef habitat type was coded as a categorical variable (fringing reef, lagoon reef, back reef, outer reef). Reef area surface was quantified in a 20-km buffer around each stereo-BRUVS using coral reef map (www.unep-wcmc.org). The depth and SST were included in the models as linear and quadratic terms to test for non-linear effects.

The level of human impact on a given reef is related to the nearby human density and its isolation from the regional capital or domestic markets^25,30,31^. Isolation was represented by the minimum travel time from each stereo-BRUVS to the capital city Noumea hosting the main market of the country. The travel time was calculated by the sum of the time needed to travel from the stereo-BRUVS to the nearest landing point in inhabited areas and the time needed to travel from this point to the capital city by road^25,26^. For stereo-BRUVS not deployed around the main island, and more than 50 km away from the first inhabited area, travel time was computed between Noumea and stereo-BRUVS by the sea. Human population density was calculated as the human population in a 25-km buffer centered at the nearest landing point from each stereo-BRUVS divided by reef area within a 20-km buffer around each stereo-BRUVS^26^ (www.insee.fr). The travel time from the capital city and the human population density were log transformed in the models to not contravene the assumptions of the subsequent models.

The level of management was split into three categories along a gradient of restrictions: open (not protected), no-take (extracting activities prohibited) and no-entry (human presence prohibited) taking advantage of 8 reserves established in New Caledonia (Table 1).

### Shark occurrence and behavior models

To investigate how isolation from humans affects the occurrence and behavior of grey reef shark, we extracted the marginal effect of the travel time from the capital city on the probability of occurrence, the probability of biting the bait and the probability of each approach type from the GLM and multinomial models using the visreg package^32^.

To evaluate the efficiency of management, we used two approaches: (i) we compared the marginal effect of travel time in the different management areas providing the net benefit of the different regulations along the isolation gradient; (ii) we extracted the fitted values of the models in the different reserves and performed pairwise permutational Student t-tests to identify their effectiveness against two extreme benchmarks (*i.e*. heavily anthropized areas and wilderness areas).

Correlation between explanatory variables were computed using Pearson, Spearman and Kendall’s indices (Supplementary Table S1). Variance inflation factors calculated for each explanatory variables in the models remained lower than 5.4, indicating that collinearity did not impair conclusions from the model outputs. Explanatory variables were selected using an automated model selection algorithm from the MuMIn package^33^ that generates a set of models with different variables combinations and ranks their importance (Supplementary Table S2).

Models accuracy was evaluated using odds ratio, kappa coefficient and Fisher’s exact test that assessed the dependence between the observed and predicted value and tested its significance. Then, the model prediction accuracy was evaluated using the confusion matrices, the sensitivity and the specificity. Each model showed odds ratio below to 1, kappa coefficient close to 1 and Fisher’s exact test p-value below 3.07 10^−6^, verifying a significant dependence between observations and predictions. Each model showed high sensitivity (ranging from 0.55 to 0.90) and specificity (ranging from 0.65 to 0.90), reducing the probability of erroneous predictions (Supplementary Table S3).

Pairwise comparisons between management categories were performed using permutational Student’s t-test from the RVAideMemoire package^34^. We used Bonferroni correction to cope with inflated type I error due to the repetition of tests. All analyses were performed using R statistical software^35^ including the libraries visreg^32^, MuMin^33^, bestglm^36^, nnet^37^, and ggplot2^38^ for models and graphic representation.

### Effect of the abundance of competitors and individual features on shark behavior

In addition to environmental and human-related variables, we evaluated the effect of the abundance of conspecifics and heterospecifics (*i.e*. other shark species) on the grey reef shark behavior. The foraging behavior of grey reef sharks is context dependent and may be modified according to the abundance of conspecifics in a given area with individuals exhibiting a bolder behavior when in groups^39,40^. The number of conspecifics and heterospecifics recorded on the videos varied from 0 to 12 and from 0 to 3, respectively, while the number of conspecifics and heterospecifics between the arrival of the first individual and the bite varied from 0 to 6 and from 0 to 3, respectively. To evaluate the effect of surrounding sharks on bite occurrence and approach type, we performed a permutational analysis of variance (PERMANOVA, 999 permutations, R statistical software) using the number of conspecifics and heterospecifics present in the video between the arrival of the first grey reef shark and the first bite. These two explanatory variables were not included in the full Generalized and Multinomial models since trivially related to isolation from humans and protection. We chose to test them apart to justify their *a priori* exclusion.

When possible, we measured the body size and sex of the first shark individuals entering the field of view to evaluate the effect of individual features on shark behavior. Body size and sex can directly influence foraging behavior through the amount of energy required per unit of body mass. It conditions prey-predator relationships by scaling mouth gape^41^. Several shifts in behavioral foraging have been documented for sharks^42,43^. Moreover, as individuals grow, they gain experience and have increased likelihood of encountering humans. Shark total length was measured for 108 individuals and ranged from 0.64 to 1.99 m (mean ± SD = 1.15 ± 0.29 m). Sex was determined for 97 individuals and the sex ratio was biased toward females (1:1.5). To evaluate the effect of these individual features on bite occurrence and approach type, we performed a permutational analysis of variance (PERMANOVA, 999 permutations). To ensure an individual response, we used data where the first shark entering the field of view was the same individual to bite the bait first (80 individuals retained). These two explanatory variables could not be included in the full Generalized and Multinomial models since only measured on a limited number of individuals. We tested their effect apart to justify their exclusion from the final models.

## Results

### Predictors of reef shark occurrence and behavior

A total of 379 grey reef sharks were recorded in the 367 stereo-BRUVS. They occurred in 140 videos (38.1% of cases). We first tested the effect of the abundance of competitors and individual features on shark behavior. Bite occurrence significantly increased when more grey reef sharks were recorded in the area (PERMANOVA, 999 permutations, p-value < 0.001, Supplementary Table S4). However, the approach type did not significantly vary with the abundance of conspecifics (p-value = 0.07). Both bite occurrence and approach type were not significantly affected by the abundance of other shark species that may be considered as competitors (p-value = 0.21 and 0.26 respectively). Considering only the surrounding competitors present when the behavior was recorded (between the first grey reef shark arrival and the first bite), the abundance of conspecifics or other shark species did not significantly affect both bite occurrence (p-value = 0.09 and 0.75 respectively) and approach type (p-value = 0.09 and 0.75 respectively). Moreover, both bite occurrence and approach type were not influenced by individual features such as body size and sex (PERMANOVA, 999 permutations, p-value > 0.32, Supplementary Table S5).

The selection procedure of explanatory variables revealed that isolation from human population, through the travel time from the capital city, was one of the main predictors of shark occurrence, bite and approach type (Supplementary Table S2). We thus focused on this predictor rather than on local population density to model the impact of human pressure. This prominent role points out that, even in absence of shark fishing, the isolation from human activities influences the probability of occurrence and the behavior of grey reef sharks. By contrast, management was only the 5^th^ and last selected variable predicting bite and approach type respectively. The importance of the other variables is presented in Supplementary Table S2.

### Increase of shark occurrence with isolation from humans and restrictions

**Figure 2:**
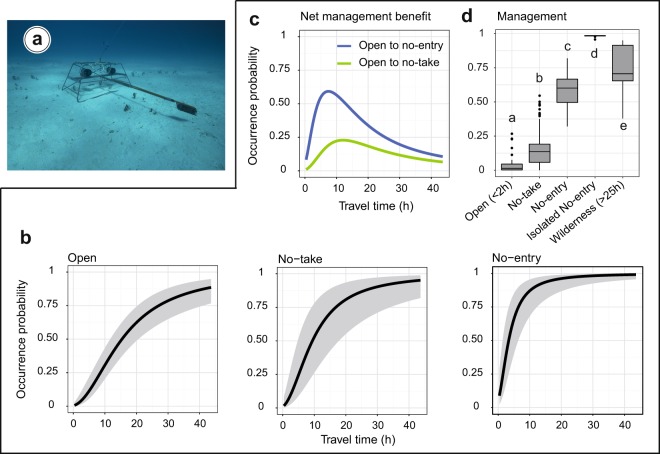
Stereo-BRUVS device (**a**) and predicted probability of occurrence for grey reef sharks as a function of travel time from the capital city across three management categories (**b**) and the management categories while accounting for other factors using linear regression models (**d**). The net protection benefit represents the difference in the predicted probability of occurrence between the different management categories (**c**). In panel (**d**), the open to fishing categories were split between areas close to humans (open with travel time shorter than 2 hours) and wilderness areas (travel time greater than 25 hours) to provide two extreme benchmarks. In the same way, the no-entry marine reserves were split with one close to humans (Merlet: No-entry) and the other isolated (Beautemps-Beaupré: Isolated No-entry). In panel (**d**), different letters indicate significant differences at p-value < 5% using permutational pairwise tests with 999 permutations.

The marginal effect of travel time from the capital city revealed a contrasted probability of grey reef shark occurrence along the isolation gradient (Fisher’s exact test p-value < 2.2 10^−16^). The predicted probability of occurrence was close to 0 near humans (low travel time) and tended to 1 when travel time was above the level of 25 hours from the capital city. This pattern was similar whatever the management but with different rates of increase. The more restrictive was the management, the higher was the increasing rate in the probability of occurrence with isolation (Fig. 2b). In wilderness areas (travel time greater than 25 hours), where there is no interaction with human activities, predicted probability of occurrence outreached 71.7%. At 2 hours away from the capital city, the predicted occurrence decreased to 3% in areas open to human activities, at 7.3% in no-take areas where human presence is allowed and at 32.1% in no-entry areas where human presence is banned. The marginal plots of the other explanatory variables are shown in Supplementary Fig. S1.

The net benefit of management on grey reef shark occurrence, obtained by comparing the predicted probability of occurrence between management categories, varied along the isolation gradient (Fig. 2c). For all reserve categories, the net protection benefit was hump-shaped tending to zero close to humans, then increased and finally decreased far from humans. It reached the highest value for no-entry marine reserves compared to areas open to fishing with an increased probability of occurrence of 59.2% at 7.3 hours away from the capital city. Meanwhile, the highest net protection benefit was of 22.9% at 12 hours away from the capital city for no-take marine reserves compared to areas open to fishing.

The predicted probability of shark occurrence varied significantly between management categories (permutational pairwise t-test, 999 permutations, p-value < 0.5%, Fig. 2d). An average probability of shark occurrence of 4.4%(±6.9% SD) was obtained in areas open to fishing close to the capital city (less than 2 hours), 16.1% (±14.4% SD) in no-take reserves, 58.4% (±12.2% SD) in the no-entry Merlet reserve, and up to 98.2% (±0.9% SD) in the isolated no-entry Beautemps-Beaupré atoll. By comparison we obtained a probability of shark occurrence of 74% (±15.1% SD) in wilderness areas.

### More bites with isolation from humans and restrictions

Bites occurred in 103 out of 140 videos (73.6% of cases) in which sharks were present. In 42% of cases, biting occurred without any prior contact with the stereo-BRUVS. In the remaining 58%, shark individuals were in contact on average 3.4 (±3.7 SD) times before biting. When no bite occurred in a video, individuals remained at an average distance of 5.4 m (±5.2 SD) from the bait.

**Figure 3:**
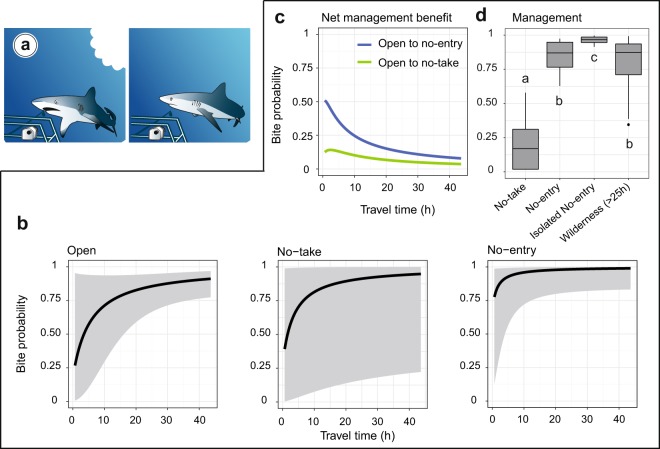
Illustration of the two bite modalities (occurrence/absence) (**a**) and predicted probability of bite of grey reef sharks on a bait as a function of travel time from the capital city across three management categories (**b**) and the management categories while accounting for other factors using linear regression models (**d**). The net protection benefit represents the difference in the predicted probability of bite between the different management categories (**c**). In panel (**d**), the lack of measurements in areas close to human population did not allow to plot the open (travel time shorter than 2 hours) modality and perform the pairwise tests. The no-entry reserves were split with one close to humans (Merlet: No-entry) and the other isolated (Beautemps-Beaupré: Isolated No-entry). Different letters indicate significant differences at p-value < 5% using permutational pairwise tests with 999 permutations. Scientific drawings courtesy of P. Lopez.

The probability of bite showed a significant marginal response with travel time from the capital city (Fisher’s exact test p-value = 6.28 10^−10^, Supplementary Table S3). The lowest probability of bite was predicted close to humans and increased towards 1 when isolation reached 10 hours of travel time. This pattern was similar across management categories but with different rates of increase. The more restrictive was the management, the higher was the rate of increase in the probability of bite (Fig. 3b). In wilderness areas, the predicted probability of bite reached 85.6%. At 2 hours away from the capital city, the few remaining grey reef sharks showed altered behaviors. The probability of bite decreased to 39.6% in areas open to fishing and to 53.4% in no-take marine reserves. Conversely, the predicted probability of bite in no-entry marine reserves located at only 2 hours away from the capital city was similar to wilderness areas (86%). The marginal plots of the other explanatory variables are shown in Supplementary Fig. S1.

The net benefit of management on the predicted probability of bite had the highest value close to humans. Near the coastline of the capital city (low travel time), establishing a no-entry marine reserve can increase the probability by 50.9% while establishing a no-take reserve would increase this probability by 12.5% only. The net benefit of management slowly decreased along the isolation gradient and was close to zero far from humans (Fig. 3c).

The predicted probability of bite was significantly different between management categories (permutational pairwise t-test, 999 permutations, p-value < 0.5%, Fig. 3d). No-take marine reserves showed a significant lower mean predicted probability of bite (20% ± 19.4% SD) while the no-entry Merlet reserve showed no significant difference with wilderness areas (p-value > 0.05) with a mean probability of 84.8% (±11.7% SD) vs. 81.2% (±15.8% SD), respectively. In the isolated no-entry Beautemps-Beaupré marine reserve almost all grey reef sharks seen on videos bit the bait (96.3% ± 3.1% SD).

### Alteration of approach type towards the bait in a human-dominated seascape

A total of 58 individuals (42.3%) adopted a straight-line approach (coded 2) showing high motivation for food intake and low cautiousness towards the stereo-BRUVS device. A total of 56 individuals (40.9%) displayed a twisted approach (coded 1) showing interest for the bait but cautiousness towards the device while 23 individuals (16.8%) did not show any attractiveness and the highest level of cautiousness (coded 0).

The marginal effect of travel time from the capital city revealed that the approach behavior towards the bait strongly varied along the isolation gradient (Fisher’s exact test p-value = 6.28 10^−10^, Supplementary Table S3). In reference conditions (*i.e*. wilderness areas at more than 25 hours away from the capital city), the predicted probability of exhibiting a straight-line approach varied between 44.7% and 73.9%. Therefore, this was the most common behavior in quasi-absence of human. Conversely, the predicted probability of not exhibiting any approach varied between 0.9% and 12.8% in the same conditions. The marginal plots of the other explanatory variables are shown in Supplementary Fig. S2.

**Figure 4:**
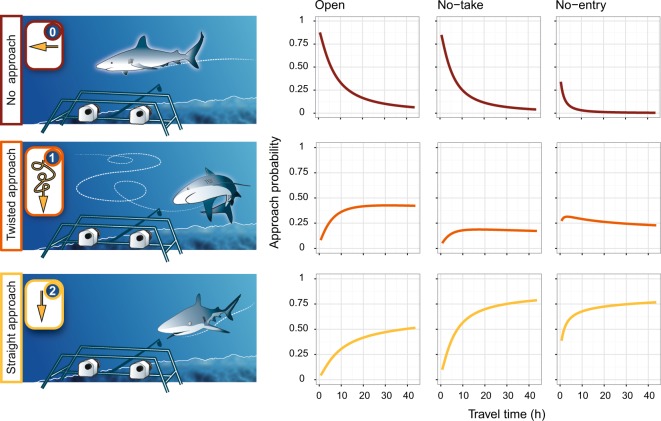
Predicted probability of the three approach types of grey reef sharks towards stereo-BRUVS as a function of the travel time from the capital city in three levels of management and accounting for other factors using multinomial regression models. Scientific drawings courtesy of P. Lopez.

Close to humans, the predicted probability of exhibiting both straight line and twisted approaches were close to 0 while the predicted probability of not approaching the bait had the highest value (84.6% in open areas, 80.6% in no-take reserves and 28.3% in no-entry reserves, Fig. 4). By contrast, in no-entry marine reserves, even close to humans, the predicted probability of exhibiting a straight line and a twisted approach reached 42.6% and 29%, respectively.

**Figure 5:**
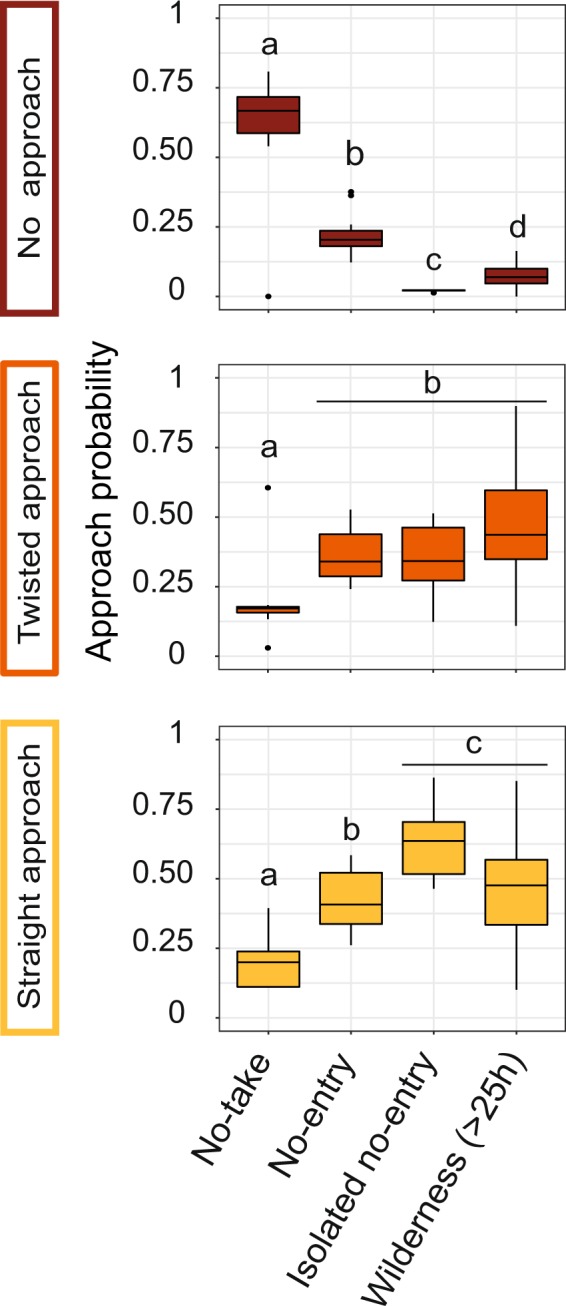
Predicted probability of the three approach types of grey reef sharks towards stereo-BRUVS as a function of the management categories while accounting for other factors using multinomial regression models. The no-entry reserves were split with one close to humans (Merlet: No-entry) and the other isolated (Beautemps-Beaupré: Isolated No-entry). Different letters indicate significant differences at p-value < 5% using permutational pairwise t-tests with 999 permutations. The lack of measurements in areas close to human population did not allow to plot the open (travel time below 2 hours) modality and perform the pairwise tests.

The comparison of the fitted values from the multinomial model revealed that the predicted probability of each approach type was significantly different between management categories (permutational pairwise t-test, 999 permutations, p-value < 0.05, Fig. 5). The mean predicted probability of exhibiting both straight line and twisted approaches were significantly the lowest in no-take marine reserves (20% ± 9.1% SD and 20% ± 15.6% SD respectively) where the mean predicted probability of not exhibiting any approach was significantly the highest (60% ± 22% SD). The predicted probability of both straight line and twisted approaches in the old large no-entry Merlet reserve and the isolated no-entry reserve of Beautemps-Beaupré were not different from wilderness areas (permutational pairwise t-test, p-value = 0.08, 999 permutations).

## Discussion

Beyond a dramatic decrease in shark occurrence, our study suggests that shark feeding behavior and reaction to novelty are altered close to humans with a marked absence of bite on the bait and more cautious approaches. In our case, unknown factors have certainly triggered a dramatic decrease in the population of grey reef sharks in a country where shark fishing is historically absent. Several hypotheses can be evoked regarding the way human-driven impacts can operate: (*i*) some human activities can simultaneously impact both shark abundance and behavior through lethal interactions such as poaching, bycatch^44^ and disturbances such as noise, photography flashes or mating disruption^45^, even though Bradley *et al*.^46^ suggest that shark behavior is not affected by SCUBA divers. Sharks are known to react to fish discarding, feeding, and the use of attractant, which may disrupt feeding behavior^47,48^. The elusive behavior in response to human activities is similar to a prey reaction to a predator encounter^49,50^. We suggest that if an individual have experienced repeated deleterious experiences when encountering human activities, an elusive behavior would be adopted when confronted to a new stimulus. In contrast, a naïve individual would favor an inquisitive behavior towards novelty. (*ii*) Some human activities can affect shark behavior by impacting their abundance. Direct removal of individuals through poaching or bycatch may have more impacted bold individuals that are likely to interact with humans than shy individuals. This may have led to an individual-based selection. Human activities may also alter the ecological niche of sharks (fish prey and coral reef habitat) which can affect their behavior. (*iii*) Some human activities can affect shark abundance by impacting their behavior. The behavioral alteration toward higher cautiousness may induce an additional energy cost that can be linked to a fitness decrease with potential consequences on population dynamics^49^. In a context of shark exploitation, overfishing is the main factor leading to population depletion^16,17,51^, but behavioral impacts may also contribute to stock collapses through a decreasing fitness. In New Caledonia, where there is no historical shark fishing, our results suggest that the behavioral alteration may indeed contribute to population decline. Additionally, our study raises questions about the kind of impacts sharks are experiencing that are inducing behavioral alterations.

Taking advantage of the unique isolation gradient and the diversity of marine reserves across the New Caledonian archipelago, we show that prohibiting extracting activities through a no-take restriction is not enough to preserve grey reef sharks. Additionally, human disturbance in no-take reserves continues to worsen with an increasing number of recreational users over the last ten years^22^. Conversely, no-entry restriction appears valuable to preserve the occurrence and the behavior of grey reef sharks. The benefit provided by this restrictive management decreases with travel time from the capital city as isolation acts as a natural protection from human impacts. Thus, the no-entry Merlet reserve (172 km²), relatively close to humans, offers the best management option for protecting shark occurrence and behavior at a relative proximity to the capital city. Although not reaching occurrence levels of wilderness areas (58.4% and 98.2% occurrence in the Merlet reserve and wilderness areas, respectively), this old, large and no-entry reserve protects an unaltered behavior of grey reef sharks and raises questions about the phenomena mitigating its benefits. By contrast, the isolated traditionally managed no-entry reserve of Beautemps-Beaupré cannot provide more protection than wilderness for reef shark occurrence and behavior. Thus, the effectiveness of reserves for sharks is conditioned by the ability to exclude human presence and avoid interactions with humans. Keeping human activities away can be achieved by setting remote reefs under protection and by enforcing strict no-entry rules. To be effective for reef sharks, reserves must also be large enough to encompass the shark home range^52^, keeping in mind that 172 km^2^ (Merlet) can be insufficient close to large human settlements. Thus, large no-entry reserves have the potential to provide efficient protection for reef sharks if they include the essential habitats for all life stages of the species^53^. When setting up such reserves, a trade-off must be reached between remoteness, which limits anthropogenic disturbance, and efficient enforcement.

Imposing themselves as apex predators, humans have led to dramatic decline of competitor species and downgrading trophic level of ecosystems^54,55^. Beyond direct overexploitation, these impacts might have acted as a selection pressure on species with behavioral plasticity^56^. The constant pressure in human-dominated areas can affect the fitness of species, leading to possible population declines. It can also produce biased censuses with risk of misinterpretations. Considering behavior in censuses is challenging but must not be ignored for species with high learning abilities. For instance, elephants are shown to travel more at night than during the day in areas with higher poaching levels^3^. With the increasing use of automated video surveys of wildlife (unmanned aerial vehicles, underwater automated vehicles) and the implementation of computer vision in ecology^57^, we strongly believe that the study of megafauna behavior is currently entering a new era and has the potential to reveal unsuspected changes in wildlife behavior under human pressure.

## Supplementary information

Supplementary information

## Data Availability

The data that support the findings of this study are available in Dryad digital repository.
