# Supplementary material for: Isolation and no-entry marine reserves mitigate anthropogenic impacts on grey reef shark behavior
Source: Sci Rep. 2019 Feb 27;9:2897. doi: 10.1038/s41598-018-37145-x (PMC6393451; doi:10.1038/s41598-018-37145-x)
Supplement: Supplementary file 1 — Supplementary information [file 41598_2018_37145_MOESM1_ESM.pdf]

Isolation and no-entry marine reserves mitigate anthropogenic impacts on grey reef shark behavior

JUHEL Jean-Baptiste, VIGLIOLA Laurent, WANTIEZ Laurent, LETESSIER Tom B., MEEUWIG Jessica J.,

MOUILLOT David

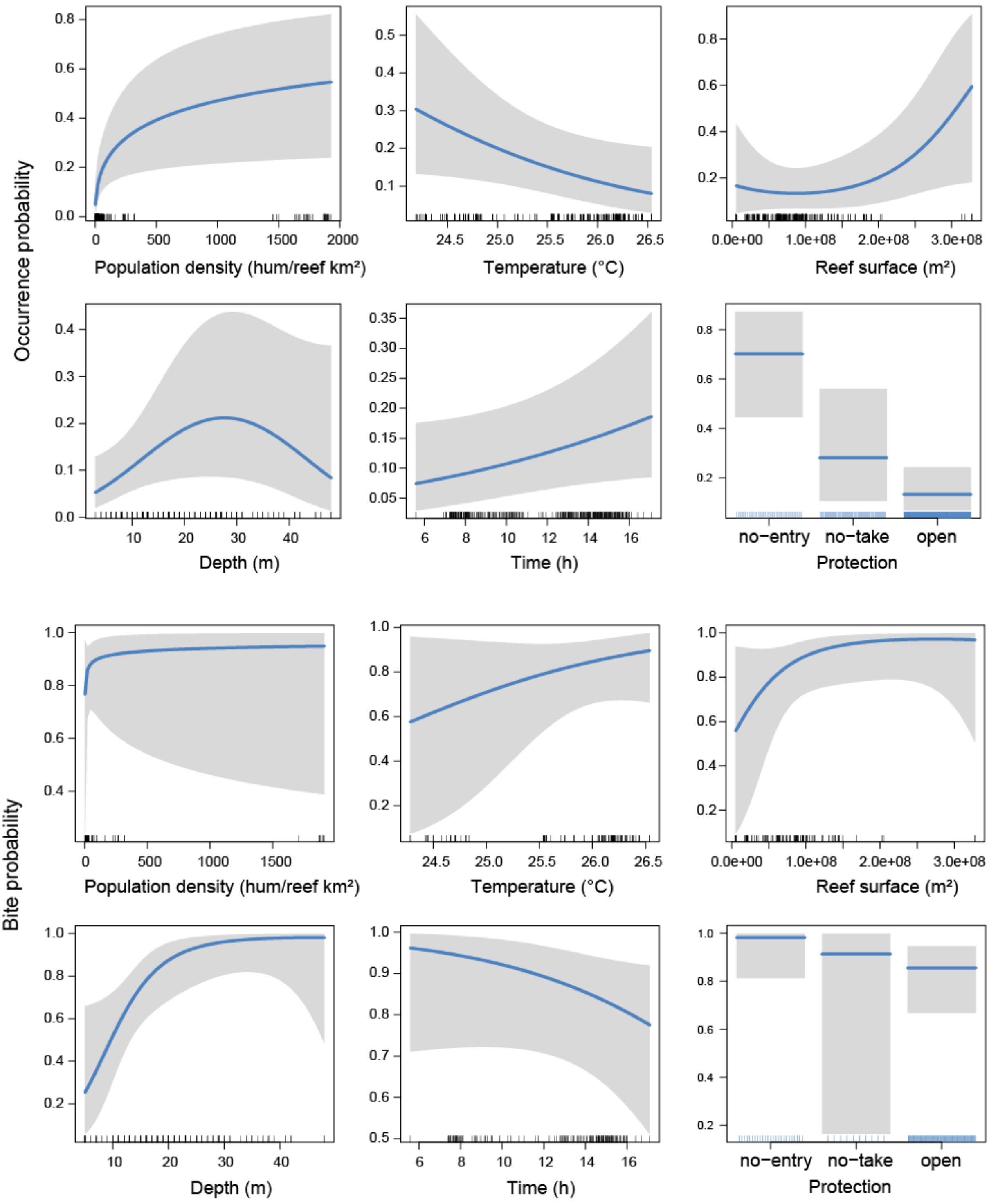

Figure S1. Marginal plots of explanatory variables from the GLM of grey reef shark occurrence and bite.

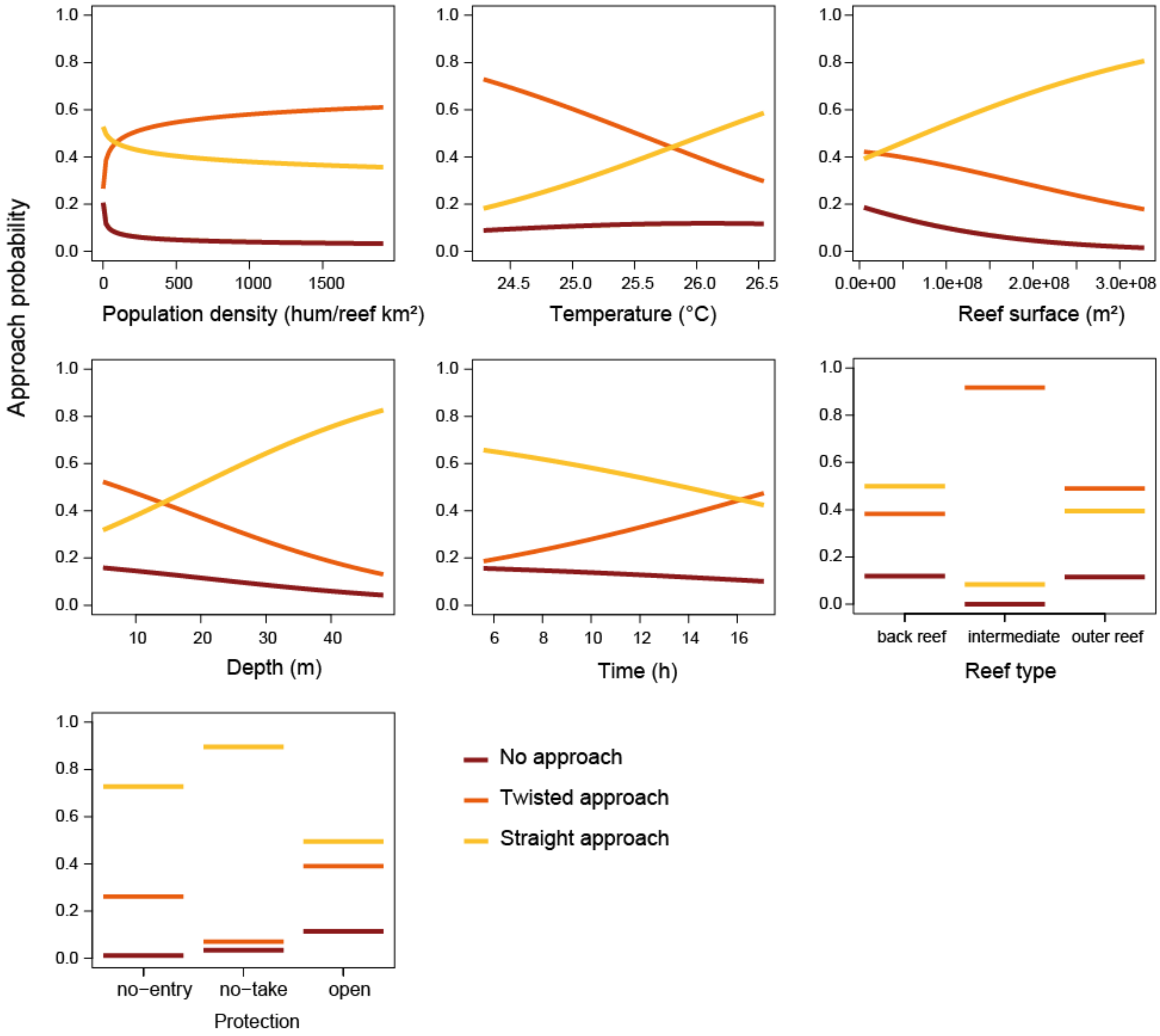

Figure S2. Marginal plots of explanatory variables from the multinomial model of grey reef shark approach type.

Table S1. Correlation coefficients between the explanatory variables used in the Generalized Linear Models (GLM) and the multinomial model.

**Pearson**	Time	Reef type	Depth	Isolation	Temperature
Reef type	-0.235				
Depth	0.072	0.331			
Isolation	0.268	-0.031	0.343		
Temperature	0.173	-0.115	0.103	0.588	
Pop. density	-0.015	0.091	-0.198	-0.315	-0.351
**Spearman**	Time	Reef type	Depth	Isolation	Temperature
Reef type	-0.198				
Depth	0.104	0.389			
Isolation	0.226	-0.037	0.345		
Temperature	0.194	-0.129	0.073	0.662	
Pop. density	0.112	0.089	-0.087	-0.445	0.057
**Kendall**	Time	Reef type	Depth	Isolation	Temperature
Reef type	-0.148				
Depth	0.072	0.311			
Isolation	0.143	-0.032	0.238		
Temperature	0.127	-0.103	0.049	0.477	
Pop. density	0.078	0.069	-0.058	-0.301	0.047

Table S2. Importance of explanatory variables based on AIC weights from the best set of models (MuMIn package, Barton 2016).

Response var.	Explanatory var.	Importance	N containing models
Occurrence	Log(Isolation +1)	1	14
	Log(Pop. density +1)	1	14
	Management	1	14
	Reef type	1	14
	Temperature	0.82	11
	Time	0.80	11
	Reef surf.	0.66	9
	Depth	0.65	10
	I($Depth^2$)	0.41	6
	I(Reef $surf.^2$)	0.12	2
Bite	Depth	1	18
	Log(Isolation +1)	0.75	13
	Reef surf.	0.63	11
	Temperature	0.25	5
	Management	0.23	4
	Log(Pop. density +1)	0.21	4
	Time	0.20	4
	I($Depth^2$)	0.15	3
	I(Reef $surf.^2$)	0.09	2
	Reef type	0.05	1
Approach type	Log(Isolation +1)	0.77	128
	Depth	0.75	128
	Reef type	0.49	128
	Temperature	0.38	128
	Time	0.30	128
	Reef surf.	0.23	128
	Log(Pop. density +1)	0.16	128
	Management	0.12	128

Table S3. Performance of the models predicting the occurrence of grey reef sharks, the bite and the approach type towards the bait. *N* represents the number of observations. For the approach type multinomial model, the modality 0 is fixed to evaluate the others. Thus the odds ratio is set at 1 for this modality.

Fitted variable	N	Sensitivity	Specificity	Kappa	Odds ratio	Odds ratio CI	Fisher's test p-value
Shark occurrence	367	0.86	0.82	0.83	26.86	14.71 - 51.21	$< 2.2\ 10^{-16}$
Bite occurrence	140	0.90	0.65	0.78	14.99	5.63 - 43.06	$6.28\ 10^{-10}$
Approach type	137	0 : 0.55	0 : 0.90	0.33	1	NA	$3.07\ 10^{-6}$
		1 : 0.59	1 : 0.69		9.83	2.60 - 43.73	
		2 : 0.59	2 : 0.74		9.81	2.08 - 61.88	

Table S4. Permutational analysis of variance (999 permutations) evaluating the effect of conspecifics and heterospecifics abundance on grey reef shark behaviour. Nb of conspecifics: MaxN of grey reef shark recorded on the video; nb of heterospecifics: MaxN of other shark species recorded on the video; nb of conspec. before bite: MaxN of grey reef shark recorded between the arrival of the first individual and the first bite; nb of heterospec. before bite: MaxN of other shark species recorded between the arrival of the first grey reef shark and the first bite

Response variable	Terms	N	D.F.	Sums of Sq.	Mean Sq.	F. Model	R^2	P-value
Approach type	Nb of conspecifics	137	1	1.748	1.748	3.362	0.024	0.068
	Nb of heterospecifics		1	0.648	0.648	1.247	0.009	0.260
	Residuals		134	69.662	0.520	0.967		
	Nb of conspec. before bite	102	1	0.9361	0.936	3.036	0.030	0.090
	Nb of heterospec. before bite		1	0.0333	0.033	0.108	0.001	0.751
	Residuals		99	30.521	0.308	0.969		
Bite	Nb of Conspecifics	140	1	4.0554	4.055	24.265	0.149	<0.001
	Nb of Heterospecifics		1	0.2694	0.269	1.612	0.010	0.213
	Residuals		137	22.897	0.167	0.841		
	Nb of conspec. before bite	102	1	0.936	0.936	3.036	0.030	0.090
	Nb of heterospec. before bite		1	0.033	0.033	0.108	0.001	0.745
	Residuals		99	30.521	0.308	0.969		

Table S5. Permutational analysis of variance (999 permutations) evaluating the effect of grey reef shark body size and sex on behaviour. *N*: number of video used in the analysis; body size of 1st shark: total length of the 1st shark entering the field of view.

Response variable	Terms	N	D.F.	Sums of Sq.	Mean Sq.	F. Model	R^2	P-value
Approach type	Body size of 1st shark	80	1	0.326	0.326	0.979	0.013	0.321
	Sex of 1st shark		1	0.111	0.111	0.333	0.004	0.564
	Body size x Sex		1	0.247	0.247	0.743	0.009	0.388
	Residuals		76	25.315	0.333		0.973	
Bite	Body size of 1st shark	81	1	0.003	0.003	0.022	<0.001	0.879
	Sex of 1st shark		1	<0.001	<0.001	0.002	<0.001	0.945
	Body size x Sex		1	0.348	0.348	2.72	0.034	0.102
	Residuals		77	9.871	0.128		0.966	
